# Diagnostik und medikamentöse Therapie der chronischen Rhinosinusitis

**DOI:** 10.1007/s00106-025-01635-y

**Published:** 2025-04-16

**Authors:** Sven Becker

**Affiliations:** Universitätsklinik für Hals-, Nasen- und Ohrenheilkunde, Kopf- und Halschirurgie, Eberhard Karls Universität Tübingen, Elfriede-Aulhorn Str. 5, 72076 Tübingen, Deutschland

**Keywords:** Nasenpolypen, Biologika, Präzisionsmedizin, Lebensqualität, Typ-2-Entzündung, Nasal polyps, Biologics, Precision medicine, Quality of life, Type 2 inflammation

## Abstract

Die chronische Rhinosinusitis stellt eine der häufigsten chronischen Erkrankungen des oberen Atemwegs dar und geht mit einer deutlichen Einschränkung der Lebensqualität einher. Eine präzise Diagnostik ist notwendig, um Patienten einer suffizienten Therapie zuführen zu können. Durch die Einführung von Biologika haben sich neue Optionen zur Behandlung schwer betroffener Patienten mit chronischer Rhinosinusitis mit Nasenpolypen im Sinne der Präzisionsmedizin ergeben, die über die alleinige Kontrolle der Symptome hinausgehen. Damit einher ging eine gewisse Standardisierung in Bezug auf diagnostische Verfahren und die Erfassung der krankheitsspezifischen Lebensqualität. Im Rahmen des vorliegenden Referats soll vor dem Hintergrund des Kongressthemas 2025 „Individualisierung vs. Standardisierung in der HNO-Heilkunde“ die Diagnostik und medikamentöse Therapie der chronischen Rhinosinusitis mit Schwerpunkt auf die Biologikatherapie beleuchtet und zusammengefasst werden.

## Abstract (en)

Chronic rhinosinusitis is one of the most common chronic diseases of the upper respiratory tract and is associated with a significant reduction in quality of life. Precise diagnostic workup is therefore necessary to provide patients with adequate treatment. The introduction of biologics has opened up new options for the treatment of severely affected patients with chronic rhinosinusitis with nasal polyps in the sense of precision medicine that goes beyond the mere control of symptoms. This has been accompanied by a certain degree of standardization with regard to diagnostic procedures and the assessment of disease-specific quality of life. Against the background of the 2025 congress theme “Individualization vs. standardization in otorhinolaryngology,” this paper examines and summarizes the diagnosis and drug therapy of chronic rhinosinusitis, with a focus on biologic therapy.

## Hintergrund

Die chronische Rhinosinusitis (CRS) ist mit einer Prävalenz von 6–11 % in der Gesamtbevölkerung eine der häufigsten chronischen Erkrankungen des oberen Respirationstrakts [1–5]. Sie ist durch eine chronische Entzündung der Schleimhäute der Nase und Nasennebenhöhlen gekennzeichnet und führt zu einer deutlichen Einschränkung der Lebens- und Schlafqualität [6–8]. Hierdurch entstehen erhebliche direkten und indirekte Gesundheitskosten [9, 10].

Die CRS ist durch typische Symptome definiert, von denen mindestens 2 länger als 12 Wochen bestehen müssen [11, 12]:

- Nasenatmungsbehinderung
- Anteriore und/oder posteriore Sekretion
- Gesichtsschmerz
- Riechstörung

Fakultativ:

- Fieber
- Kopfschmerz

Neben der Anamnese ist ein pathologischer Befund in der Rhinoskopie/nasalen Endoskopie oder der Bildgebung durch Computertomographie (CT), digitale Volumentomographie (DVT) oder – seltener – Magnetresonanztomographie (MRT) erforderlich. Nur eine Endoskopie kann das Vorhandensein von Polypen bestätigen, was folgende Einteilung ermöglicht:

- *C*hronische *R*hino*s*inusitis mit (englisch „*w*ith“) *N*asen*p*olypen (*CRSwNP*)
- *C*hronische *R*hino*s*inusitis ohne (lateinisch „*s*ine“) *N*asen*p*olypen (*CRSsNP*) [12]

Im European Position Paper on Rhinosinusitis and Nasal Polyps von 2020 (EPOS2020) wird die CRS anhand von 3 Aspekten weiter klassifiziert [4, 13]:

- Ist die Erkrankung primär oder sekundär zu einer anderen zugrunde liegenden Erkrankung zu sehen?
- Ist die Erkrankung beidseits diffus vorliegend oder lokalisiert auf eine Seite oder Nebenhöhle?
- Welches Entzündungsmuster (Endotyp) liegt der Erkrankung vermutlich zugrunde?

Das vorliegende Referat bezieht sich auf die primäre, beidseits diffus vorliegende CRS, die im Folgenden vereinfacht als CRS bezeichnet wird.

**Abb. 1:**
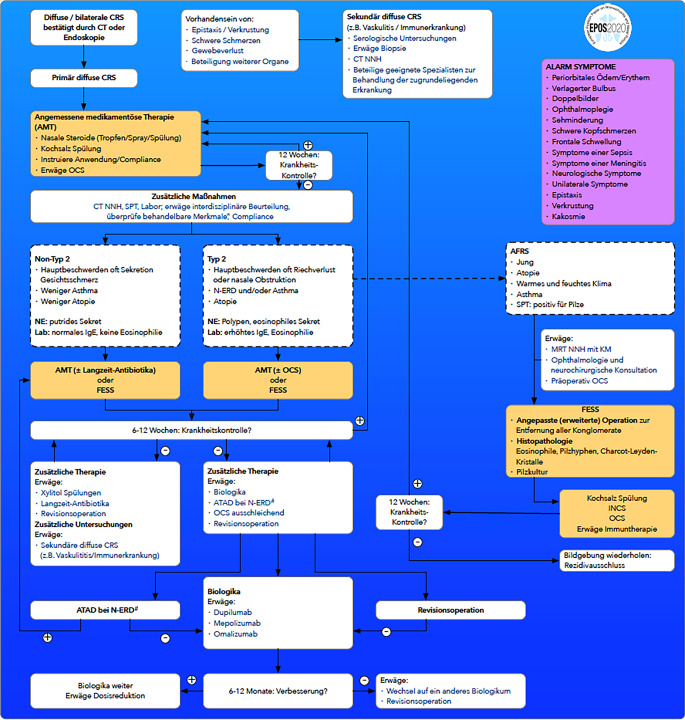
Behandlungspfad zum Management der primären diffusen chronischen Rhinosinusitis (CRS) [5]. *AFRS* „allergic fungal rhinosinusitis“, allergische Pilzrhinosinusitis;* AMT* angemessene medikamentöse Therapie; *ATAD* „aspirin-therapy after desensitization“, Aspirin-Dauertherapie nach adaptiver Desaktivierung; *CRS* chronische Rhinosinusitis; *CT* Computertomographie; *EPOS* European Position Paper on Rhinosinusitis and Nasal Polyps; *FESS* funktionelle endoskopische Nasennebenhöhlenchirurgie; *IgE* Immunglobulin E; *INCS* intranasale Kortikosteroide; *KM* Kontrastmittel; *Lab* Labor; *MRT* Magnetresonanztomographie; *NE* Nasenendoskopie; *NERD* durch NSAID exazerbierte Atemwegserkrankung; *NNH* Nasennebenhöhlen; *NSAID* nichtsteroidale Antirheumatika; *OCS* orale Kortikosteroide; *SPT* „skin prick test“, Hautpricktest. *Asterisk* Behandelbare Merkmale: Rauchen, Allergie, Beruf und Defizite der mukoziliären Clearance. *Raute* Eine Behandlung mit Aspirin nach adaptiver Desaktivierung (ATAD) sollte nur bei NERD-Patienten in Betracht gezogen werden, bei denen keine Kontraindikationen für die langfristige Einnahme hoher Aspirindosen vorliegen. (Mod. nach [13]). (Danksagung an Prof. M. Wagenmann für die deutsche Übersetzung)

Nach der Diagnosestellung zählen zur Standardtherapie der CRS neben der Nasendusche mit diversen Zusätzen intranasale Glukokortikosteroide (nGKS) in Form von Nasensprays oder Spülungen. Die Edukation des Patienten hat ebenfalls einen wichtigen Stellenwert (Abb. 1; [4, 13]). Bleiben die Symptome nach 12 Wochen bestehen und ist eine entsprechende Pathologie in der Bildgebung nachgewiesen, können weiterführende Untersuchungen zur Abschätzung des zugrunde liegenden Endotyps durchgeführt werden.

Diese beinhalten z. B. die allergologische Abklärung mittels Hautpricktest, spezifischem Immunglobulin E (IgE) und Gesamt-IgE, laborchemische Untersuchungen mittels Differenzialblutbild zur Erhebung der Eosinophilenzahl, die Verlaufsbeurteilung der Beschwerden sowie der rhinoskopischen/endoskopischen Befunde [4, 13].

Anhand der beschriebenen Diagnostik können grob 2 zugrunde liegende Entzündungstypen unterschieden werden, die als Typ-2-Inflammation und Nicht-Typ-2-Inflammation bezeichnet werden. Erstere geht meist mit einem Riechverlust und nasaler Obstruktion einher. Es finden sich häufig Atopien, Asthma oder eine durch nichtsteroidale Antirheumatika (NSAID) exazerbierte Atemwegserkrankung (N-ERD, Samter-Trias, Widal-Trias). Die Endoskopie zeigt Polypen, und laborchemisch können erhöhte Werte für Eosinophile und Gesamt-IgE gemessen werden [4, 13].

Bei Patienten mit einer Nicht-Typ-2-Inflammation stehen die anteriore und/oder posteriore Rhinorrhoe sowie Gesichtsschmerzen im Vordergrund. Asthma und Atopien finden sich weniger häufig. Die Endoskopie zeigt selten Polypen, sondern eine teilweise stark gerötete Schleimhaut mit Eiterstraßen. Ein Hinweis auf eine Sensibilisierung oder erhöhte Gesamt-IgE und Eosinophilenwerte ergibt sich meist nicht [4, 13].

Die Typisierung hilft bei der Verlaufsprognose und Therapieentscheidung (ggf. Langzeitantibiotika bei Nicht-Typ-2-Inflammation, systemische Glukokortikosteroide [sGKS] bei Typ-2-Inflammation). Sind konservative Maßnahmen ausgereizt, ist eine funktionelle endoskopische Nasennebenhöhlenoperation (FESS) angezeigt. Patienten mit Typ-2-Inflammation weisen dabei ein höheres Risiko für Rezidive nach FESS auf.

Ist die Chirurgie nicht in der Lage, eine ausreichende Symptomlinderung zu generieren, stehen für die Typ-2-Inflammation seit 2019 Biologika zur Behandlung einer schweren CRSwNP zur Verfügung. Die vormals häufiger durchgeführte Aspirin-Dauertherapie nach adaptiver Desaktivierung (ATAD) bei N-ERD sowie eine dauerhafte oder repetitive Einnahme von sGKS haben dadurch nur noch in Einzelfällen ihre Bedeutung.

Für Patienten mit rezidivierenden Beschwerden bei Nicht-Typ-2-Inflammation sind Biologika im Rahmen von Studien in klinischer Erprobung. Die Langzeitgabe von Antibiotika (Clarithromycin, Erythromycin, Roxithromycin) kann erwogen werden [14–17]. Zur Lokaltherapie kann in die Nasendusche Xylitol aufgenommen werden.

Das tiefere Verständnis der Pathophysiologie der CRS mit den verschiedenen Endotypen hat die Einführung von Biologika ermöglicht. Diese erlauben es, im Sinne der Präzisionsmedizin in den Entzündungsprozess einzugreifen [18]. Die Präzisionsmedizin strebt eine individualisierte Behandlung auf Basis biologischer, genetischer und umweltbezogener Merkmale an [19]. Auch wenn belastbare Biomarker zur Auswahl des entsprechenden Biologikums aktuell noch fehlen, war die Einführung in die Rhinologie ein Meilenstein in der Behandlung schwer betroffener CRSwNP-Patienten.

Im Rahmen des vorliegenden Referats werden vor dem Hintergrund des Kongressthemas 2025 „Individualisierung vs. Standardisierung in der HNO-Heilkunde“ die Diagnostik und medikamentöse Therapie der CRS mit Schwerpunkt auf der Biologikatherapie beleuchtet und zusammengefasst.

## Pathophysiologie

**Abb. 2:**
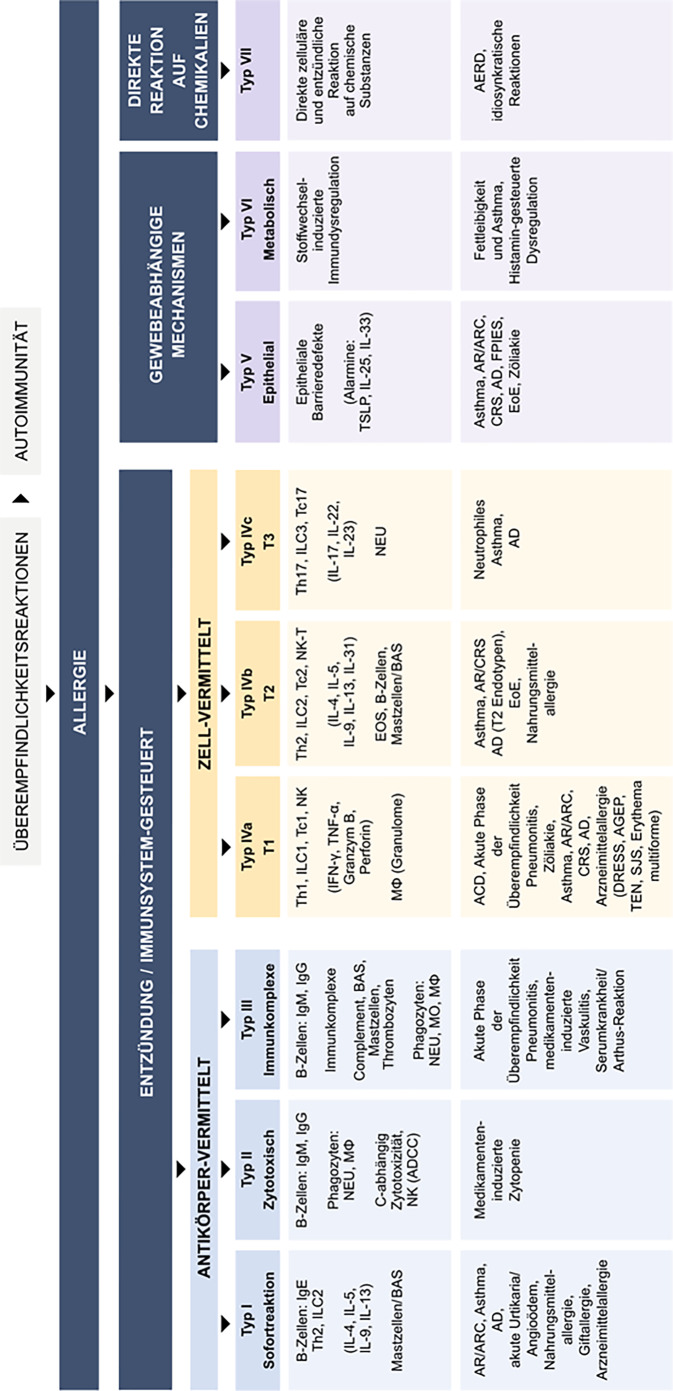
Neue Nomenklatur allergischer Erkrankungen. Unter Überempfindlichkeit versteht man eine unerwünschte, unangenehme oder schädliche Reaktion, die durch eine Dysfunktion der Gewebezellen oder eine Überreaktion des Immunsystems entsteht. Eine Allergie ist eine überschießende Reaktion auf exogene Reize, die verschiedene Arten von Überempfindlichkeitsreaktionen umfasst, an denen Antikörper, immunzellvermittelte, gewebegesteuerte oder metabolische Mechanismen beteiligt sind und die zur Entwicklung von Atemwegs‑, Haut‑, Augen‑, Magen-Darm- und anderen Symptomen, einschließlich Anaphylaxie, führen. *ACD* allergische Kontaktdermatitis; *AD* atopische Dermatitis; *ADCC* antikörperabhängige zelluläre Zytotoxizität; *AERD* Aspirin-exacerbated respiratory disease; *AGEP* akute generalisierte exanthematische Pustulose; *AR* allergische Rhinitis; *ARC* allergische Rhinokonjunktivitis; *B* B-Lymphozyten; *BAS* Basophile; *CRS* Chronische Rhinosinusitis; *DRESS* schwere Arzneimittelreaktion mit Eosinophilie und systemischen Symptomen; *EoE* eosinophile Ösophagitis; *EOS* Eosinophile; *FPIES* nahrungsproteininduziertes Enterokolitissyndrom; *IFN‑γ* Interferon-gamma; *Ig (E,* *G, M)* Immunglobulin (Typ E, G, M); *IL* Interleukin; *ILC1/2/3* „innate lymphoid cells“ vom Typ 1/2/3; *MO* Monozyten; *Mφ* Makrophagen; *NEU* Neutrophile; *NK* natürliche Killerzelle; *NK‑T* natürliche Killer-T-Zelle; *SJS* Stevens-Johnson-Syndrom; *T1/T2/T3* Typ 1/2/3 Immunreaktion; *Tc1/2/17* zytotoxische T‑Lymphozyten Typ 1/2/17; *TEN* toxische epidermale Nekrolyse; *Th* T-Helfer-Lymphozyten; *TSLP* thymic stromal lymphopoietin; *TNF‑α* Tumornekrosefaktor-alpha. (Aus [197], mod. nach [28])

Der technologische Fortschritt in molekularer Diagnostik, Omics-Technologien, Genetik und Epigenetik, Nanotechnologie sowie bei bildgebenden Verfahren und vielem mehr hat ein tieferes Verständnis pathophysiologischer Mechanismen ermöglicht. Diese Erkenntnisse führten zur Definition immunologischer Endotypen und erforderten eine Überarbeitung der Nomenklatur allergischer Erkrankungen und Hypersensitivitätsreaktionen [20–27]. Die European Academy of Allergy and Clinical Immunology (EAACI) hat 2023 basierend auf der Coombs-und-Gell-Klassifikation eine neue Einteilung in 9 immunologische Reaktionstypen vorgeschlagen, die aktuelles Grundlagenwissen integriert und eine Fortentwicklung in der Zukunft ermöglicht (Abb. 2; [28]).

Die CRS findet sich sowohl unter den zellvermittelten Entzündungen im Sinne einer Reaktion vom Typ IVa/b/c als auch bei den gewebevermittelten Epithelbarrieredefekten als Typ-V-Reaktion wieder. Als Reaktion auf chemische Substanzen wird sie unter den Typ-VII-Reaktionen subsumiert. Die Typ-IVb-Reaktion, bisher als Typ-2- oder T2-Endotyp bezeichnet, war in den vergangenen Jahren aufgrund der Einführung von Biologika zunächst im Bereich der atopischen Dermatitis sowie des Asthmas, später im Bereich der CRS Gegenstand intensiver Forschungsbemühungen.

### Typ-2-/Typ-IVb-Inflammation

Die Typ-2-Inflammation (Typ IVb nach neuer Nomenklatur [n. n. N.]) liegt in westlichen Industrienationen in 90 % der Fälle allein oder als Mischtyp der CRSwNP zugrunde. Auf zellulärer Ebene spielen Typ-2-polarisierte CD4+-T-Helfer-Zellen (Th2-Zellen), innate Lymphozyten der Klasse 2 (ILC2), Eosinophile sowie Makrophagen und natürliche Killer-T-Zellen (NK-T-Zellen) eine wichtige Rolle [28]. Die Entzündungsreaktion ist dabei primär gegen große extrazelluläre Parasiten gerichtet. Th2-Zellen produzieren große Mengen an Interleukin (IL)-4, IL‑5, IL-13, aber auch IL‑9, IL-31 und Eotaxine. Schlüsselzytokin ist IL‑4, welches für einen Klassenwechsel in der Immunglobulinsynthese in B‑Zellen weg von Immunglobulin (Ig) M und IgG hin zu IgE verantwortlich ist [29]. IL-13 führt über einen Gewebeumbau zur Chronifizierung der Erkrankung. IL‑5 vermittelt die Ausreifung und Freisetzung von Eosinophilen aus dem Knochenmark ins Blut, deren Rekrutierung aus dem Blut in das jeweilige Entzündungsgebiet sowie deren Überleben im entzündeten Gewebe selbst. Die Degranulation von Eosinophilen führt zur Freisetzung von toxischen Substanzen wie z. B. eosinophiles kationisches Protein (ECP), „major basic protein“ (MBP) oder eosinophiler Peroxidase, die das Gewebe nachhaltig schädigen (Remodelling) und so zur Chronifizierung der Erkrankung beitragen [30, 31]. Neben den Th2-Zellen spielen auch die ILC2-Zellen eine zentrale Rolle bei der Orchestrierung der Typ-2-Inflammation. Wie erstgenannte Zellen, mit denen sie in Wechselwirkung stehen, produzieren sie ebenfalls IL‑4, IL‑5 und IL-13. Sie reagieren stark auf aus dem Epithel ausgeschüttete Alarmine wie IL-33, IL-25 und „thymic stromal lymphopoetin“ (TSLP) und wirken über IL-13 öffnend auf die epitheliale Barriere zurück [32, 33]. Hier überlappen die Entzündungsmechanismen der Typ-2-Inflammation (Typ IVb n. n. N.) mit den Mechanismen, die sich bei der Typ-V-Reaktion zumindest in Bezug auf das Asthma finden [32]. Durch Inhibition von Gewebe-Plasminogen-Aktivatoren (t-PA) kommt es zu einer Deposition von Fibrinnetzen, die die Gewebematrix für die Polypen darstellen [4].

### Typ-1-/Typ-IVa-Inflammation

Die Typ-1-Inflammation (Typ IVa n. n. N.) wird durch ein anderes Zell- und Interleukinmuster (T1-Endotyp) charakterisiert und führt phänotypisch meist nicht zur Ausbildung von Polypen, sondern verursacht Beschwerden im Sinne einer CRSsNP. Auf zellulärer Ebene sind Typ-1-polarisierte T‑Helfer-Zellen (Th1-Zellen) sowie innate Lymphozyten der Klasse 1 (ILC1) von Bedeutung, die große Mengen an Interferon‑γ (IFN-γ), Lymphotoxin und Tumornekrosefaktor‑α (TNF-α) produzieren. Der Entzündungsmechanismus zielt dabei auf eindringende virale Pathogene. Durch die Synthese von IgG1 und IgG3 durch B‑Zellen und die Aktivierung zytotoxischer T‑Zellen werden Krankheitsmechanismen, wie z. B. die Ausbildung von Granulomen, vermittelt [34]. Die Aktivierung von Neutrophilen führt im längeren Verlauf zu einer Schädigung der Epithelbarriere, welche das Eindringen weiterer Pathogene erleichtert [4].

### Typ-3-/Typ-IVc-Inflammation

Die Typ-3-Inflammation (Typ IVc n. n. N.) beruht zellulär auf der Aktivierung von Th17-Zellen und ILC3, die v. a. an der Produktion von IL-17 und IL-22 beteiligt sind [35]. Die Entzündungsreaktion ist gegen extrazelluläre Bakterien und Pilze gerichtet und geht weniger häufig mit endonasalen Polypen einher. Wie bei der Typ-1-Inflammation (Typ IVa n. n. N.) kommt es zu einer starken Aktivierung von Neutrophilen, die die Epithelbarriere schädigen und das Eindringen weiterer Pathogene erleichtern [4].

### Typ-V-Inflammation

Neben den zellvermittelten Entzündungsreaktionen ist seit Jahren auch die Epithelbarriere Gegenstand intensiver Forschungsbemühungen, um mukosale und kutane Erkrankungen wie allergische Rhinitis, allergisches Asthma, CRS, aber auch die atopische Dermatitis sowie die eosinophile Ösophagitis besser verstehen zu können. In vielen Fällen scheint dabei der Verlust der Barrierefunktion eine wichtigere Rolle zu spielen als eine primäre zellvermittelte Immundysregulation [36]. Nach neuer Nomenklatur werden gewebevermittelte epitheliale Immunreaktionen als Typ-V-Reaktionen beschrieben (Abb. 2; [28]). Die eingeschränkte Barrierefunktion erleichtert hierbei die Aktivierung des Immunsystems und führt bei längerfristigem Bestehen zu einer chronischen Entzündung. Die Schädigung des Epithels kann dabei von außen durch unterschiedliche Pathogene und Schadstoffe verursacht werden [37, 38]. Die Ausschüttung von Alarminen wie „thymic stromal lymphopoetin“ (TSLP), IL-33 und IL-25 aus dem geschädigten Epithel kann dann die bereits genannten Entzündungsmechanismen, v. a. aber eine Typ-2-Inflammation (Typ IVb n. n. N.) in Gang setzen [39]. Dieser Entzündungstyp kann durch hohe IL-13-Spiegel direkt negativen Einfluss auf die „tight junctions“ der Epithelzellen haben und die Barrierestörung unterhalten und vorantreiben [33, 40]. Biologika mit dem Ziel, Alarmine wie TSLP oder IL-33 zu blockieren, sind für das Asthma bereits teilweise zugelassen und werden in Zukunft wahrscheinlich auch für die CRSwNP zur Verfügung stehen [41].

### Typ-VII-Inflammation

Bei der Typ-VII-Reaktion n. n. N. kommt es zu einer direkten zellulären und inflammatorischen Antwort auf chemische Substanzen (Abb. 2; [28]). Ein Beispiel stellt die N-ERD dar. Charakterisiert wird die Erkrankung durch eine Trias aus Asthma, wiederkehrender CRSwNP und Überempfindlichkeit gegenüber Acetylsalicylsäure (Aspirin) oder anderen NSAID [42]. Zugrunde liegt der Erkrankung ein Ungleichgewicht im Arachidonsäurestoffwechsel, der zur Überproduktion von Leukotrienen und Verringerung antiinflammatorischer Prostaglandine führt [43]. Acetylsalicylsäure und andere NSAID inhibieren die Zyklooxygenase, genauer die beiden Isoenzyme COX‑1 und COX‑2, welche an der Synthese von Leukotrienen und Prostaglandin beteiligt sind. Dies führt zur Verstärkung des bereits bestehenden Ungleichgewichts und resultiert in einer Zunahme der Entzündungsreaktion mit Bronchokonstriktion, erhöhter vaskulärer Permeabilität, Sekretproduktion und Rekrutierung weiterer Entzündungszellen im oberen und unteren Atemweg [28]. Die starke Aktivierung von Th2-Zellen mit Ausschüttung von Typ-2-Zytokinen wie IL‑4, IL-13 und IL‑5 führt zu einer längerfristigen eosinophilen Entzündung im Gewebe [44]. Alle aktuell in Deutschland zugelassenen Biologika konnten aufgrund der teilweise zugrunde liegenden Typ-2-Inflammation (Typ IVb n. n. N.) ihre Wirksamkeit beim Vorliegen einer AERD unter Beweis stellen [45–48].

Aufgrund der besseren Lesbarkeit sowie der bisher nur langsamen Verbreitung der vorgestellten neuen Nomenklatur wird im Folgenden nur die herkömmliche Terminologie verwendet. Typ 1 entspricht dabei dem Typ IVa, Typ 2 dem Typ IVb und Typ 3 dem Typ IVc.

## Diagnostik

### Anamnese

Die Anamnese sollte neben der Abfrage der für die Diagnosestellung wichtigen Symptome ein umfängliches Bild des Patienten und seiner Beschwerden ergeben. Dies beinhaltet weitere Symptome und Diagnosen im HNO-Bereich, die Medikamentenanamnese v. a. in Bezug auf die Einnahme von Kortisonpräparaten, die Erfassung von Allergien und Komorbiditäten (v. a. Asthma), vorangegangene Operationen (v. a. Nasennebenhöhlen[NNH]-Operationen) sowie Fragen nach dem Arbeitsumfeld. Der Anamnese schließt sich die Erhebung eines kompletten HNO-Status an.

### Klinische Untersuchung

#### Polypen-Score

Die Diagnose einer CRS basiert neben der Anamnese auf der Beurteilung des Naseninneren, im Optimalfall durch eine nasale Endoskopie der anterioren und posterioren Nasenabschnitte im unteren, mittleren und oberen Nasengang. Nur durch die Rhinoskopie/Endoskopie kann eine Aussage über das Vorhandensein von nasalen Polypen getroffen werden und eine Einteilung der Erkrankung in CRSsNP und CRSwNP vorgenommen werden. Eine solche Unterscheidung allein anhand der Bildgebung ist nicht möglich!

**Abb. 3:**
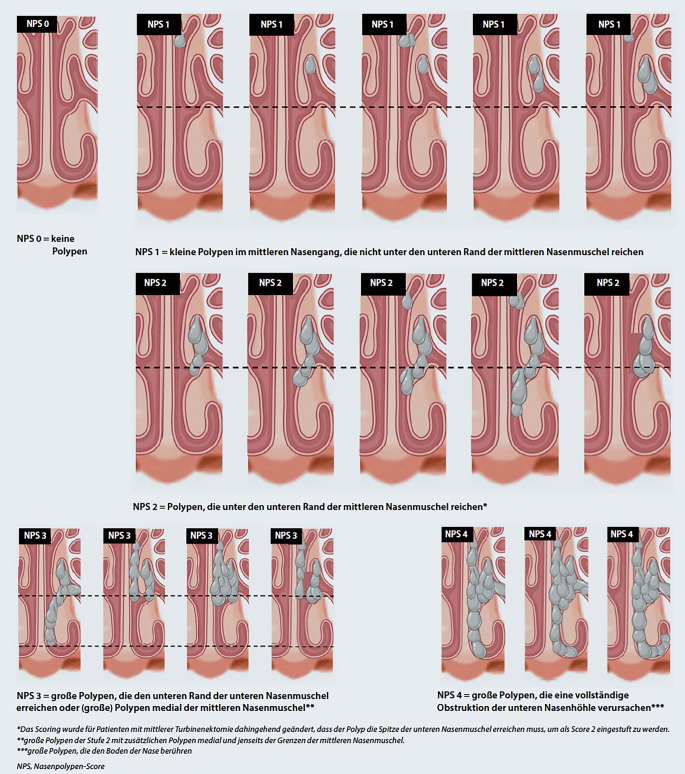
Visuelle Darstellung des Nasenpolypen-Score. (Aus [198], mod. nach [49])

Während sich für die CRSsNP bisher kein Scoring-System zur Beurteilung des endonasalen Befundes international durchsetzen konnte, hat sich zur Beurteilung der CRSwNP ein Scoring-System etabliert [49]. Dieses fand bei der Durchführung unterschiedlicher Phase-II- und -III-Studien zu Biologika als primärer Endpunkt Anwendung [50–55]. Es kann heute als Standardmethode zur Beurteilung endonasaler Polypen bezeichnet werden. Die Tab. 1 gibt Aufschluss über die Art der Bewertung, Abb. 3 visualisiert die Befunde schematisch.

**Tab. 1:** Nasenpolypen-Score zur Dokumentation des nasalen Endoskopiebefundes bei Chronischer Rhinosinusitis mit Nasenpolypen (CRSwNP)

Nasenpolypen-Score	Polypengröße
**0**	*Keine Polypen*
**1**	*Kleine Polypen im mittleren Nasengang, die nicht unter die untere Grenze der mittleren Nasenmuschel reichen*
**2**	*Polypen, die unter den unteren Rand der mittleren Nasenmuschel reichen (Das Scoring wurde für Patienten, bei denen eine Resektion/Teilresektion der mittleren Nasenmuschel durchgeführt wurde, dahingehend geändert, dass der Polyp die Spitze der unteren Nasenmuschel erreichen muss, um als Score 2 eingestuft zu werden)*
**3**	*Große Polypen, die den unteren Rand der unteren Nasenmuschel erreichen, oder Polypen, die medial der mittleren Nasenmuschel liegen (großer Polyp der Stufe 2 mit zusätzlichen Polypen medial und jenseits der Grenzen der mittleren Nasenmuschel)*
**4**	*Große Polypen, die eine vollständige Obstruktion der unteren Nasenhöhle verursachen (große Polypen, die den Nasenboden berühren)*

Allgemeine Regel: Eine Punktzahl kann nur zugewiesen werden, wenn die Kriterien der niedrigeren Punktzahl erfüllt sind

(Aus [198], mod. nach [49])

Der Score beschreibt die Ausprägung der Befunde und kann bei korrekter Anwendung optimal zum Therapiemonitoring im klinischen Alltag herangezogen werden.

#### HNO-Status

Neben der Rhinoskopie/Endoskopie sollte ein komplettierender HNO-Status (Ohrmikroskopie, Untersuchung der Mundhöhle, Laryngo‑/Pharyngoskopie und Palpation des Halses) erhoben werden, um weitere Erkrankungen im HNO-Bereich auszuschließen. Bei der Ohrmikroskopie kann sich der Hinweis auf eine weitere, durch Eosinophile vermittelte Typ-2-Erkrankung ergeben, die eosinophile Otitis media [56].

### Erfassung der Lebensqualität: Skalen und Scores

#### Sino-Nasal Outcome Test 22

Um die Lebensqualität erfassen zu können, sind neben allgemeinen Lebensqualitätsfragebögen, wie z. B. dem Short Form(SF)-36 krankheitsspezifische, auf die Erkrankung oder das Organsystem abgestimmte Fragebögen notwendig. Einer der am häufigsten bei CRS-Patienten eingesetzten Fragebögen ist der Sino-Nasal Outcome Test 22 (SNOT-22) [57]. Er wurde für den englischen Sprachraum entwickelt und dafür auch validiert, liegt in der Zwischenzeit aber für 16 weitere Sprachen in validierter Form vor. Die deutsche Version wurde von der Arbeitsgemeinschaft Rhinologie (ARHIN) der Deutschen Gesellschaft für Hals‑, Nasen- und Ohrenheilkunde, Kopf- und Hals-Chirurgie sowie einer weiteren Arbeitsgruppe validiert [58, 59].

Der Fragebogen umfasst 22 Symptome, die in 4 Subskalen (physische Symptome, emotionale und psychische Symptome, funktionale Beeinträchtigungen und weitere Symptome) unterteilt werden können. Jedes Item quantifiziert den Schweregrad des Symptoms auf einer Skala von 0 (kein Problem) bis 5 (maximale Beeinträchtigung), mit einer maximalen Punktzahl des Fragebogens von 110 [58].

Der Fragebogen ist ein etabliertes Instrument zur Verlaufsbeurteilung, insbesondere unter Biologikatherapie, und ermöglicht die objektive Erfassung der Lebensqualitätsveränderung. Die „minimal important difference“ (MID) oder auch „minimal clinically important difference“ (MCID) wurde in der englischen Validierungsstudie mit 8,9 Punkten definiert, was einer klinisch relevanten Verbesserung oder Verschlechterung entspricht [57]. Wie der Polypen-Score wurde auch der SNOT-22 bei allen Zulassungsstudien der aktuell zugelassenen Biologika als Endpunkt verwendet [50, 54, 60].

Weitere weniger häufig im Bereich der CRS eingesetzte Fragebögen sind z. B. der Rhinosinusitis Disability Index (RSDI), der Fragebogen Nasal Obstruction Symptom Evaluation (NOSE) oder der Rhinoconjunctivitis Quality of Life Questionnaire (RQLQ) bei eher allergologischen Fragestellungen [61–65].

#### Visuelle Analogskalen

Neben strukturierten Fragebögen sind visuelle Analogskalen (VAS) ein effektives Werkzeug zur schnellen und zuverlässigen Erfassung subjektiver Symptome. Sie können Veränderungen im Zustand eines Patienten präzise und schnell über die Zeit erfassen und sind damit für Klinik und Forschung optimal geeignet. Patienten quantifizieren ihre Symptome auf einer 10-cm-Skala (0 = Symptomfreiheit, 10 = stärkste Symptomausprägung) [66]. Häufig verwendete VAS bei CRS sind:

- Gesamtsymptomlast
- Nasale Obstruktion
- Rhinorrhoe/postnasaler Drip
- Gesichtsschmerz/Druck
- Riechverlust
- Schlafstörungen

Das EPOS2012 führte erstmals Kriterien zur Krankheitskontrolle der CRS ein [67]. Diese wurden in mehreren Studien untersucht und validiert [68–70]. Im EPOS2020 wurde die Notwendigkeit der Evaluation der Krankheitskontrolle bekräftigt [4]. Zur Beurteilung der Kontrolle der CRS in „kontrolliert“, „teilweise kontrolliert“ und „unkontrolliert“ gehen neben den 5 letztgenannten VAS der endoskopische Befund sowie die Anwendung von oralen Kortikosteroiden ein.

#### Asthma Control Test

Das häufig gemeinsame Auftreten von CRS und Asthma erfordert die regelmäßige Erfassung des Asthmastatus insbesondere bei Biologikatherapien, da diese sich auch meist positiv auf den unteren Atemweg auswirken [71].

Der Asthma Control Test (ACT) dient der Beurteilung der Asthmakontrolle anhand von 5 Fragen, die sich jeweils auf die letzten 4 Wochen beziehen und mit 1–5 Punkten (1 = stärkste Ausprägung, 5 = keine Symptome) bewertet werden [72]:

1. Wie oft hatten Sie Atembeschwerden?
2. Wie oft sind Sie wegen Ihrer Asthmasymptome aufgewacht oder mussten früh aufstehen?
3. Wie stark haben Asthmasymptome Ihre üblichen Aktivitäten (zu Hause, in der Arbeit oder in der Freizeit) eingeschränkt?
4. Wie oft haben Sie Ihre Bedarfsmedikamente verwendet?
5. Wie bewerten Sie die Asthmakontrolle?

Die Interpretation bezieht sich auf den Gesamtscore:

- 25 Punkte: voll kontrolliertes Asthma
- 20–24 Punkte: gut kontrolliertes Asthma
- Unter 20 Punkte: nicht ausreichend kontrolliertes Asthma

Ein ACT-Score unter 20 sollte eine pneumologische Abklärung veranlassen. Der ACT eignet sich zur Verlaufskontrolle bei CRSwNP-Patienten mit komorbidem Asthma unter Biologikatherapie und ermöglicht die Beurteilung der Therapieeffekte auf den unteren Atemweg.

### Weitere Diagnostik

#### Computertomographie der Nasennebenhöhlen

Neben der nasalen Endoskopie sollte zur Diagnosesicherung der CRS eine hochauflösende CT der NNH (Schichtdicke 0,5–1 mm) in den Ebenen axial, koronal und sagittal durchgeführt werden, um entzündliche Veränderungen auch in endoskopisch nicht einsehbaren Bereichen zu beurteilen [4, 12]. Alternativ kann eine DVT der NNH erfolgen.

Der Lund-Mackay-Score dient als standardisiertes Instrument zur radiologischen Quantifizierung der Verschattung der NNH und spiegelt die Entzündungslast wider [73–75].

Beurteilt werden der/die:

- Sinus maxillaris,
- Sinus ethmoidalis anteriores,
- Sinus ethmoidalis posteriores,
- Sinus frontalis,
- Sinus sphenoidalis,
- ostiomeatale Komplex

jeweils rechts und links mit einem Punktwert von:

- 0 Punkte: keine Verlegung
- 1 Punkt: teilweise Verlegung
- 2 Punkte: komplette Verlegung.

Der ostiomeatale Komplex kann lediglich die Ausprägung 0 Punkte (keine Verlegung) sowie 2 Punkte (Verlegung) annehmen. Es ergibt sich somit ein Score von minimal 0 und maximal 24 Punkten (12 Areale á maximal 2 Punkte). Der Score korreliert meist nicht mit der Einschränkung der Lebensqualität, z. B. gemessen im SNOT-22, eignet sich jedoch sehr gut zur Verlaufsbeobachtung der Erkrankung. Darüber hinaus bildet der CT-Befund die Basis für das Ausmaß einer möglichen Operation [74].

#### Hautpricktest

IgE-vermittelte Soforttypreaktionen wie die allergische Rhinitis stellen die klassische Typ-I-Reaktion nach Combs und Gell dar (Abb. 2), laufen aber längerfristig über ähnliche Interleukine und Zelltypen ab wie die CRSwNP. Sie werden damit ebenfalls zu den Typ-2-Erkrankungen (Typ IVb n. n. N.) gezählt [28]. Bei Patienten mit CRSwNP finden sich häufig weitere Komorbiditäten aus der Gruppe der Typ-2-Erkrankungen wie allergische Rhinitis oder Asthma. Der Nachweis einer allergischen Sensibilisierung mittels Hautpricktest („skin-prick test“) oder Nachweis von allergenspezifischem IgE im Serum kann daher sinnvoll zur Einordnung des zugrunde liegenden Endotyps herangezogen werden. Das Vorliegen einer entsprechenden Sensibilisierung beweist nicht eine durch eine Typ-2-Inflammation ausgelöste CRSwNP, macht diese jedoch wahrscheinlicher [4].

#### Sniffinʼ Sticks

Patienten mit CRSwNP klagen häufig über Einschränkungen des Riechvermögens bis hin zur Anosmie [4, 76]. Zur Erfassung und Verlaufskontrolle des Riechvermögens eignen sich standardisierte Tests. Im deutschsprachigen Raum eignen sich hierfür die Sniffinʼ Sticks (Fa. Burghart Messtechnik, Holm, Deutschland) [77, 78]. Während die gesamte Testbatterie einen Schwellen‑, Diskriminations- und Identifikationstest umfasst, eignet sich für die tägliche Praxis der Identifikationstest mit 12 oder 16 Riechstiften, die der Patient anhand von jeweils 4 Auswahlmöglichkeiten richtig benennen soll. Für ersteren Test ist eine Unterscheidung in eingeschränktes und normales Riechvermögen möglich. Für letztgenannten Test ist die Unterscheidung in eine Anosmie und Hyposmie möglich. Die Sniffinʼ Sticks eignen sich sowohl zur Indikationsstellung als auch hervorragend zur Verlaufsbeurteilung einer Biologikatherapie.

#### Differenzialblutbild und Immunglobulin E

Optimale Biomarker zur Beurteilung der CRSwNP und zur Vorhersage des Therapieansprechens sind derzeit weder im Serum noch im Nasensekret oder Bürstenabstrich etabliert und werden weiterhin erforscht. Für die Routinediagnostik werden jedoch die Eosinophilenzahl im Differenzialblutbild und das Gesamt-IgE im Serum herangezogen [3, 4].

Eine spezifische IgE-Diagnostik (spezifisches IgE, sIgE) auf Aeroallergene kann bei zusätzlich vorliegendem Verdacht auf eine Sensibilisierung/Allergie ebenfalls sinnvoll sein und orientiert sich an den Beschwerden des Patienten und/oder den Ergebnissen aus dem Hautpricktest [79].

#### Lokale Entzündungsmarker/Mikrobiom

Validierte lokale Biomarker für die Indikation und Verlaufsbeobachtung der CRS fehlen bislang. Auch die optimale Entnahmemethode ist noch nicht geklärt. Nasensekretproben mit Auffangschwämmchen ermöglichen die Analyse von Mediatoren wie eosinophiles kationisches Protein (ECP), Tryptase und Interleukinen (z. B. IL-5), während Bürstenabstriche oder das Abschaben von Epithelschichten zytologische Auswertungen und die Sichtbarmachung von Zellen erlauben. Diese Verfahren ergänzen Biopsien der Nasenschleimhaut [80, 81]. Auch die Untersuchung des Nasenmikrobioms könnte künftig zum besseren Verständnis der CRS-Pathogenese beitragen [82].

## Therapie

### Glukokortikosteroide

#### Topisch/intranasal

Die Anwendung von intranasalen/topischen Glukokortikosteroiden (nGKS) via Nasenspray stellt die Erstlinientherapie bei der Behandlung der CRS auf allen Versorgungsebenen dar [4, 12]. Verschiedene Reviews und eine Cochrane-Datenbank-Analyse bestätigen die gute Wirksamkeit von nGKS v. a. bei der CRSwNP [83–85]. nGKS haben einen positiven Einfluss auf die krankheitsspezifische sowie die generelle Lebensqualität bei CRS und sind auch bei Langzeitanwendung effektiv und sicher [4].

Die Zugabe von GKS zur Nasenspülung oder die Verwendung in einem Verneblersystem ist möglich. Über den „off-label use“ sollte mit dem Patienten gesprochen werden und dies in der Akte dokumentiert sein [12, 84, 86, 87].

Die topische Applikation von GKS über „drug-eluting stents“, die in den meisten Fällen im Anschluss an eine NNH-Operation eingebracht werden, kann eine Option für bestimmte Patienten sein. Größere Studien zur Langzeittherapie mit entsprechenden Sicherheitsdaten fehlen jedoch bisher [4].

#### Systemisch/oral

Die kurzzeitige Anwendung von systemischen Glukokortikosteroiden (sGKS) über 7–21 Tage ist v. a. bei CRSwNP weit verbreitet. Sie führt durch eine Unterdrückung der Entzündung zu einer schnellen Reduktion der Polypengröße und einer schnellen Verbesserung des Riechvermögens. Eine Symptomverbesserung kann bis zu 3 Monate nach Beginn der Einnahme nachgewiesen werden. Während es zu sGKS und CRSwNP mehrere doppel-blind placebo-kontrollierte Studien (DBPCT) gibt, fehlen diese zur CRSsNP [4, 88].

Nebenwirkungen einer kurzfristigen sGKS-Therapie umfassen Schlafstörungen, gastrointestinale Beschwerden sowie Stimmungsschwankungen [89]. Die häufige oder langfristige Anwendung von sGKS erhöht jedoch das Risiko schwerwiegender Nebenwirkungen. Dazu zählen u. a. Osteoporose, Hyperglykämie, Diabetes mellitus, avaskuläre Nekrosen des Hüftkopfs, gastrointestinale Beschwerden wie peptische Ulzera, kardiovaskuläre Ereignisse sowie die Ausbildung eines M. Cushing. Das Risiko solcher Nebenwirkungen steigt mit höheren Dosen und längerer Anwendungsdauer, weshalb diese Therapieform so kurz wie möglich gehalten werden sollte [90]. Der Einsatz von Biologika kann die Notwendigkeit für sGKS deutlich reduzieren [91, 92].

### Nasenspülung

Nasenspülungen gehören wie die Anwendung von nGKS zur Erstlinientherapie der CRS. Sie entfernen zähen Mukus und Krusten, führen zu einer verbesserten mukoziliären Clearance und können auch kleinere Pathogene aus der Nase entfernen. Fragen nach dem richtigen Volumen, der Temperatur sowie möglichen Zusätzen zur Spülung sind in einzelnen Studien untersucht worden, aber bisher nicht abschließend beantwortet. Die aktuelle Studienlage wurde im EPOS2020 aufgearbeitet und bewertet. Die Autoren halten eine Nasenspülung mit isotoner Kochsalzlösung oder Ringer-Laktat-Lösung für sinnvoll. Eine Zugabe von Xylitol, Natriumhyaluronat (Hyaluronsäure) oder Xyloglucan kann weitere positive Effekte haben [4].

### Antibiotika-Langzeitanwendung

Die Anzahl an Studien zur antibiotischen Langzeittherapie > 4 Wochen bei CRS sind überschaubar und qualitativ eingeschränkt [15, 93, 94]. Zusätzlich ist diese Therapieform aufgrund der Einführung der Biologika weiter in den Hintergrund getreten. Die aktuell gültige Leitlinie der Arbeitsgemeinschaft der Wissenschaftlichen Medizinischen Fachgesellschaften (AWMF) empfiehlt bei Versagen der Standardtherapie bei CRSsNP einen längerdauernden Einsatz von Clarithromycin (z. B. 500 mg/Tag für 2 Wochen, danach 250 mg/Tag für weitere 6 Wochen) zu erwägen. Im Einzelfall können auch Erythromycin und Roxithromycin eingesetzt werden. Zur Behandlung einer Rezidivpolyposis bei CRSwNP kann eine längerdauernde Therapie mit Doxycyclin (z. B. Einnahme über 3 Wochen, Tag 1: 200 mg, dann 100 mg/Tag) erwogen werden. Topische Anwendungen von Antibiotika sollten nicht erfolgen [12].

### Aspirin-Dauertherapie nach adaptiver Desaktivierung

Auch die Aspirin-Dauertherapie nach adaptiver Desaktivierung (ATAD) hat ihren Stellenwert in der Behandlung von Patienten mit N-ERD eingebüßt, da alle Phase-III-Studien der bisher für die CRSwNP zugelassenen Biologika eine gute oder sogar überdurchschnittliche Wirksamkeit in der Subgruppe der eingeschlossenen NERD-Patienten zeigen [50, 54, 60]. Bei der ATAD soll durch die wiederholte Applikation von Acetylsalicylsäure eine Toleranz gegenüber Analgetika, insbesondere COX-1-Inhibitoren, induziert werden [12]. Die Studienlage zur ATAD ist sowohl in Bezug auf die Diagnosestellung als auch auf die Therapie überschaubar. DBPCT mit großer Patientenzahl und unterschiedlichen Erhaltungsdosen im Vergleich fehlen. Eine Zulassung für die Anwendung von Acetylsalicylsäure in der Indikation N-ERD besteht nicht. In DBPCT mit unterschiedlichen Erhaltungsdosen von 100–1300 mg und geringer Patientenzahl ließ sich eine Wirkung auf den nasalen Symptomenscore und die krankheitsspezifische Lebensqualität nachweisen [95–98].

Nebenwirkungen der ATAD werden in zwischen 0 und 34 % der Fälle beschrieben und beziehen sich in vielen Fällen auf den Gastrointestinaltrakt [42].

### Weitere medikamentöse Ansätze

#### Dekongestiva

Topische Dekongestiva tragen über ihren vasokonstriktorischen Effekt zur Reduktion des nasalen Schleimhautödems bei [99]. Eine längerfristige Einnahme > 7 Tage kann jedoch zu einer Rhinitis medicamentosa führen, die zu einer Zunahme der Symptome nach Absetzen führt [100]. Der Einsatz von Dekongestiva wird bei CRS daher nicht empfohlen [4, 12].

#### Antihistaminika

Die Evidenz zur Wirksamkeit von Antihistaminika (oral oder topisch) bei CRS ist schlecht, so gibt es lediglich eine DBPCT zu Cetirizin bei Patienten mit CRSwNP [101]. Die über 3 Monate mit 45 Patienten durchgeführte Studie ergab eine Besserung des nasalen Niesreizes sowie der Rhinorrhoe zu allen Zeitpunkten. Sie zeigte jedoch keinen Einfluss auf die Polypengröße und den totalen Symptom-Score [102]. Aufgrund der schlechten Datenlage spielen Antihistaminika zur Behandlung der CRS keine Rolle, haben aber bei Patienten mit komorbiden Allergien und entsprechenden Symptomen ihre Berechtigung.

#### Leukotrienrezeptorantagonisten

Auch die Evidenz zur Wirksamkeit von Leukotrienrezeptorantagonisten bei CRS ist aufgrund der sehr geringen Anzahl an hochwertigen Studien niedrig [103]. Leukotriene sind inflammatorische Mediatoren, welche von Eosinophilen und Mastzellen durch den Abbau von Arachidonsäure synthetisiert werden können, und spielen v. a. bei der Typ-VII-Inflammation (Abb. 2) eine wesentliche Rolle [28]. Eine aktuellere Studie, die eine Add-on-Therapie von Montelukast zu nGKS postoperativ im Rahmen einer prospektiven randomisierten klinischen Studie bei 72 Patienten mit CRSwNP untersucht hat, kommt zu dem Schluss, dass eine zusätzliche Gabe von Montelukast postoperativ nicht empfehlenswert erscheint [104]. Basierend auf der vorliegenden Evidenz empfiehlt auch die Steuerungsgruppe des EPOS2020 keine zusätzliche Anwendung von Montelukast zu nGKS bei CRS [4].

### Operative Therapie

Führen die genannten Therapieoptionen, v. a. nGKS und Nasenspülungen, zu keiner für den Patienten akzeptablen Reduktion der Symptome, so ist nach weiterer Diagnostik mittels bildgebender Verfahren eine endoskopische Nasennebenhöhlenoperation (ESS) angezeigt [4, 12]. Diese erfolgt in den meisten Fällen als funktionelle endoskopische Nasennebenhöhlenchirurgie (FESS), kann in Einzelfällen aber auch darüber hinaus gehen [105]. Operative Therapieoptionen bei CRS werden an dieser Stelle nicht weiter vertieft.

### Biologika

#### Indikationsstellung

Seit November 2019 stehen Biologika für die Behandlung von Patienten mit schwerer CRSwNP zur Verfügung, die mit konservativer und operativer Therapie nicht ausreichend behandelt werden können. Biologika sind monoklonale Antikörper, die im Sinne der Präzisionsmedizin gezielt einzelne Interleukine oder deren Rezeptoren blockieren und damit Entzündungskaskaden unterbrechen können. Die zugelassenen Präparate (Dupilumab, Omalizumab, Mepolizumab) wirken ausschließlich auf die Typ-2-Entzündungskaskade und greifen an unterschiedlichen Stellen in diese ein. Biologika sind mit durchschnittlichen Jahrestherapiekosten von 15.000–20.000 € im Vergleich zu konservativen oder chirurgischen Ansätzen sehr teuer. Ihre Anwendung sollte daher aktuell nur den schwer betroffenen Patienten vorbehalten bleiben, bei denen andere Therapieformen unzureichend sind.

**Abb. 4:**
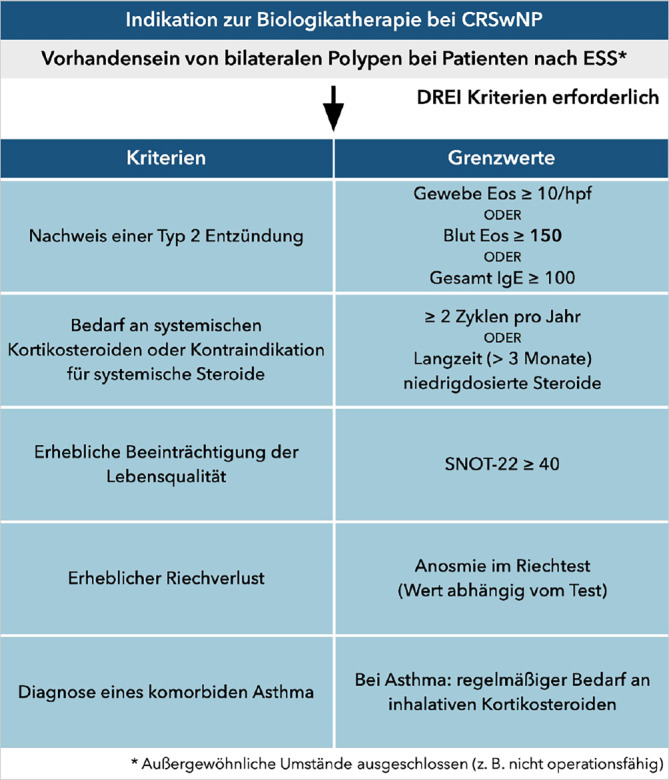
Indikationskriterien zur Biologikatherapie bei chronischer Rhinosinusitis mit Nasenpolypen (CRSwNP). *Eos* Eosinophile; *ESS* endoskopische Nasennebenhöhlenoperation; *hpf* Hauptgesichtsfeld („high-power field“) bei 400-facher Vergrößerung; *IgE* Immunglobulin E; *SNOT-22* Sino-Nasal Outcome Test 22. (Mod. nach [107]). (Danksagung an Prof. M. Wagenmann für die deutsche Übersetzung)

Zur Indikationsstellung wurden von internationalen Fachgesellschaften Empfehlungen entwickelt [4, 106]. Diese wurden im Verlauf angepasst und überarbeitet [107]. Eine Biologikatherapie ist indiziert, wenn bei einem Patienten mit beidseitiger CRSwNP nach vorangegangener Operation mindestens 3 von 5 definierten Kriterien erfüllt sind. Bei Patienten, die aufgrund physischer oder psychischer Komorbiditäten nicht operiert werden können, kann auf die operative Voraussetzung verzichtet werden. (Abb. 4).

1. Evidenz für das Vorliegen einer Typ-2-Inflammation durch Nachweis entweder einer erhöhten Eosinophilenzahl in einem histopathologischen Präparat einer vorangegangenen Operation mit mehr als 10 Eosinophilen pro Hauptgesichtsfeld („high-power field“) bei 400-facher Vergrößerung, den Nachweis einer absoluten Eosinophilenzahl im Differenzialblutbild von mehr als 150 Zellen/μl oder den Nachweis eines Gesamt-IgE-Spiegels von mehr als 100 IU/ml
2. Notwendigkeit der Anwendung von sGKS oder Kontraindikation gegen sGKS. Als relevant werden ≥ 2 mehrtägige Anwendungen von sGKS pro Jahr oder eine Langzeitanwendung dieser über mehr als 3 Monate angesehen
3. Signifikante Einschränkung der Lebensqualität mit einem Wert im SNOT-22 von ≥ 40 Punkten
4. Signifikante Einschränkung des Riechvermögens mit Nachweis einer Anosmie in einem validierten Riechtest
5. Diagnose eines Asthmas mit regelmäßiger Einnahme eines inhalativen GKS

**Abb. 5:**
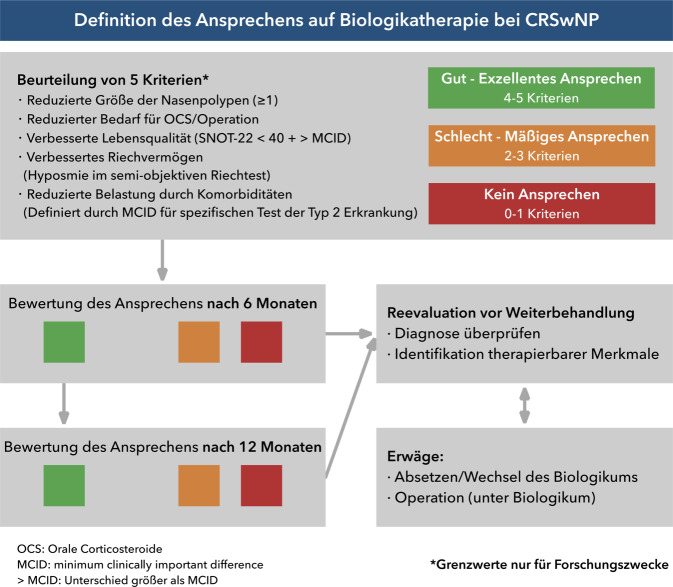
Definition des Ansprechens nach European Forum for Research and Education in Allergy and Airway Diseases (EUFOREA)/European Position Paper on Rhinosinusitis and Nasal Polyps (EPOS) auf eine Biologikatherapie bei chronischer Rhinosinusitis mit Nasenpolypen (CRSwNP) 2023. *SNOT-22* Sino-Nasal Outcome Test 22. (Mod. nach [108]). (Danksagung an Prof. M. Wagenmann für die deutsche Übersetzung)

Eine Verlaufsbeurteilung sollte sich nach 6 Monaten und 12 Monaten Therapie anschließen, um die Wirksamkeit der Therapie beurteilen zu können ([108]; Abb. 5). Die Beurteilung stützt sich dabei auf die Evaluation von 5 Kriterien.

1. Reduktion der endonasalen Polypengröße (Polypen-Score)
2. Reduzierter Bedarf von systemischen Glukokortikosteroiden/Operation
3. Verbesserung der Lebensqualität (SNOT-22)
4. Verbesserung des Riechvermögens (validierter Riechtest)
5. Reduzierte Belastung durch Komorbiditäten

Ergibt sich unter Therapie eine Verbesserung in 4–5 Kriterien, gilt das Ansprechen als gut bzw. exzellent. Bei positiver Änderung in 2–3 Kriterien wird von einem schlechten bzw. mäßigen Ansprechen ausgegangen. Bleiben die Kriterien unverändert oder zeigt sich lediglich eine Verbesserung in einem Kriterium, ist von einem fehlenden Ansprechen auszugehen. Für Forschungszwecke wurden Grenzwerte zur Beurteilung der Kriterien formuliert (Abb. 5; [108]).

Bei schlechtem bzw. fehlendem Ansprechen sollte vor Weiterbehandlung die Diagnose reevaluiert werden. Bei Fortbestehen der Indikation kann auf ein anderes Biologikum gewechselt oder eine erneute Operation (ggf. unter Biologikatherapie) durchgeführt werden. Eine erneute Verlaufskontrolle sollte sich nach weiteren 6 Monaten anschließen [107, 108].

Aktuell sind in Deutschland 3 Präparate für die Behandlung der schweren CRSwNP zugelassen [109]. Neben der richtigen Indikationsstellung ist die richtige Dokumentation wichtig, um etwaigen Regressandrohungen entgegentreten zu können [110].

#### Dupilumab

Dupilumab war 2019 das erste in Deutschland zur Behandlung der schweren CRSwNP zugelassene Biologikum [50]. Die Wirksamkeit von Dupilumab bei Typ-2-Erkrankungen wurde davor bei anderen Indikationen wie der atopischen Dermatitis sowie bei Asthma nachgewiesen [111–113]. Die Zulassungsstudien lieferten umfangreiche Sicherheitsdaten mit wenigen unerwünschten Arzneimittelwirkungen. Für die CRSwNP wurden in den Zulassungsstudien am häufigsten unerwünschte Ereignisse wie Nasopharyngitis, Erythem an der Injektionsstelle, Konjunktivitis, Keratitis, Husten, Bronchitis und Arthralgien beobachtet [50]. Nach Markteinführung wurden Arthralgien mit in die Liste der Nebenwirkungen aufgenommen [114]. Darüber hinaus zeigte sich durchgängig ein vorübergehender Anstieg der Eosinophilen im Blut [115]. Dieses Phänomen wird möglicherweise durch eine fehlende Rekrutierung von Eosinophilen aus dem Blut in das entsprechende Entzündungsgebiet verursacht [116]. Wegen der Entstehung einer möglichen Hypereosinophilie halten mehrere Autoren eine Differenzialblutbildbestimmung zu Beginn der Therapie für angezeigt [117, 118]. Der Autor des vorliegenden Referats hält eine routinemäßige Bestimmung nach 3 und 6 Monaten für sinnvoll.

Bei Dupilumab handelt es sich um einen humanen monoklonalen IgG4-Antikörper, welcher an die IL-4-Rezeptoruntereinheit α bindet. Diese findet sich sowohl im IL-4-Rezeptor als auch im IL-13-Rezeptor, sodass eine Blockade sowohl des IL-4- als auch des IL-13-Signalwegs möglich wird. Dies führt zu einer Hemmung nachgeschalteter zytokininduzierter Reaktionen, von Chemokinen und der IgE-Produktion. Eine durch die Blockade des IL-4-Signalwegs vermittelte Wiederkehr des Riechvermögens ist bei vielen Patienten das erste sich positiv ändernde Symptom und kann häufig schon nach den ersten Applikationen verzeichnet werden [119].

Dupilumab ist zugelassen als Add-on-Therapie zu intranasalen Kortikosteroiden bei erwachsenen Patienten im Alter von ≥ 18 Jahren mit schwerer CRSwNP, deren Erkrankung mit systemischen Kortikosteroiden und/oder chirurgischem Eingriff nicht ausreichend kontrolliert werden kann. Die Dosierung beträgt 300 mg Dupilumab alle 2 Wochen s.c. mittels Fertigspritze oder Fertig-Pen.

Unterschiedlichen Studien konnten die gute Wirksamkeit von Dupilumab zur Behandlung der CRSwNP auch unter Real-World-Bedingungen über unterschiedlich lange Zeiträume nachweisen [119–123]. Die Studien zeigen ein vergleichbar gutes Ansprechen wie in den Zulassungsstudien mit einer schnellen deutlichen Besserung aller Symptome innerhalb der ersten 6 Monate. Auch nach dieser Zeit kommt es mit zunehmender Therapiedauer zu einer weiteren Verbesserung der Symptomatik für einen sehr großen Anteil der Patienten.

Erste Vergleichsstudien zwischen Dupilumab und endoskopischer Nasennebenhöhlenoperation belegen eine gute Wirksamkeit beider Verfahren, zeigen jedoch eine stärkere Symptomreduktion für die mit Dupilumab behandelten Patienten nach 12 Monaten [124, 125].

Eine Verlängerung des Therapieintervalls auf > 2 Wochen scheint bei sehr gut auf die Therapie ansprechenden Patienten nach 24 Wochen problemlos möglich zu sein [126]. Aufgrund des „off-label use“ kann dies jedoch aktuell in Deutschland aufgrund der Regressgefahr nicht empfohlen werden, auch wenn hiermit deutliche Einsparungen für das Gesundheitssystem zu erzielen wären.

Zur Indikationsstellung und Verlaufsdokumentation stehen auf Dupilumab angepasste Dokumentationsbögen zur Verfügung [110, 114]. Ein Zusatzdokument zur in Überarbeitung befindlichen AWMF-Leitlinie Rhinosinusitis beinhaltet einen produktneutralen Indikationsbogen zur Biologikatherapie [5]. Zur richtigen Kodierung der CRSwNP in der Internationalen statistischen Klassifikation der Krankheiten und verwandter Gesundheitsprobleme, 10. Revision, German Modification (ICD-10-GM) vor Einleitung einer Biologikatherapie wurden ebenfalls Empfehlungen formuliert [127].

#### Omalizumab

Omalizumab ist das zweite in Deutschland für die CRSwNP zugelassene Biologikum, welches seit 2020 zur Verfügung steht [54]. Omalizumab ist ein humanisierter monoklonaler Antikörper, der gegen frei zirkulierendes IgE gerichtet ist und die Bindung des IgE an seinen Rezeptor verhindert. Neben der deutlichen Reduktion des freien IgE führt Omalizumab konsekutiv zu einer Reduktion der IgE-Rezeptordichte auf Mastzellen, Basophilen und dendritischen Zellen [128, 129].

Omalizumab ist zugelassen als Add-on-Therapie zu intranasalen Kortikosteroiden bei erwachsenen Patienten im Alter von ≥ 18 Jahren mit schwerer CRSwNP, deren Erkrankung durch eine Therapie mit nGKS nicht ausreichend kontrolliert werden kann. Die Dosis von Omalizumab richtet sich nach dem Basiswert des Gesamt-IgE sowie dem Körpergewicht des Patienten [110]. Die entsprechende Gesamtinjektionsmenge, die alle 2 oder 4 Wochen appliziert werden sollte, wird mit den entsprechenden Injektionsdosen zusammengestellt. Es stehen Fertigspritzen und Fertig-Pens mit 75 mg, 150 mg und 300 mg zur Verfügung.

Omalizumab erhielt 2003 die Zulassung für das schwere allergische Asthma in den USA [130]. Im Jahr 2013 folgte die Zulassung für die chronische spontane Urtikaria und 2020 für die CRSwNP [54, 131]. In den USA ist Omalizumab seit Februar 2024 auch für die Behandlung von Nahrungsmittelallergie zugelassen [132]. Der genaue Wirkmechanismus von Omalizumab ist bisher nicht abschließend verstanden, geht aber weit über eine bloße Bindung der freien IgE-Antikörper hinaus [133]. Aufgrund der langjährigen Anwendung stehen umfangreiche Sicherheitsdaten zur Verfügung. Fälle von Anaphylaxie nach Anwendung von Omalizumab wurden in einem sehr kleinen Anteil von Patienten (etwa 0,1–0,2 %) beschrieben, ein erhöhtes Risiko besteht v. a. dann, wenn in der Vergangenheit bereits eine Anaphylaxie unabhängig von Omalizumab aufgetreten war [134]. Gute Sicherheitsdaten liegen dagegen in der Schwangerschaft vor, die auch in einer prospektiven Studie untersucht wurden [135].

Die gute Wirksamkeit bei CRSwNP konnte aufbauend auf die Zulassungsstudien in einer Open-Label-Extensionsstudie sowie unter Real-World-Bedingungen bestätigt werden [119, 136–138].

Hilfestellung zur Indikation und Verlaufsdokumentation geben auf Omalizumab abgestimmte Dokumentationsbögen [110, 139].

#### Mepolizumab

Mepolizumab wurde 2021 als drittes Biologikum zur Behandlung der schweren CRSwNP in Deutschland zugelassen [60]. Seine Wirksamkeit auf Typ-2-vermittelte eosinophile Erkrankungen wurde zunächst für das schwere eosinophile Asthma nachgewiesen, worauf 2015 die globale Zulassung in dieser Indikation folgte [140, 141]. Die klinischen Phase-III-Studien zu Asthma, CRSwNP, Hypereosinophiliesyndrom und zur eosinophilen Granulomatose mit Polyangiitis (EGPA) zeigten ein konsistentes Sicherheitsprofil mit Reduktion der Eosinophilenwerte. Häufige unerwünschte Arzneimittelwirkungen waren Nasopharyngitis, Kopfschmerzen, Epistaxis und Rückenschmerzen [60].

Mepolizumab ist ein humanisierter monoklonaler Antikörper gegen freies IL‑5. Er verhindert dessen Bindung an den IL-5-Rezeptor auf Eosinophilen, Granulozyten, Mastzellen und weiteren Zielzellen und blockiert so gezielt die eosinophile Entzündung [142].

Mepolizumab ist zugelassen als Add-on-Therapie zu intranasalen Kortikosteroiden bei erwachsenen Patienten im Alter von ≥ 18 Jahren mit schwerer CRSwNP, deren Erkrankung mit systemischen Kortikosteroiden und/oder chirurgischem Eingriff nicht ausreichend kontrolliert werden kann. Die Dosierung beträgt 100 mg Mepolizumab alle 4 Wochen s.c. Die Applikation kann über eine 100-mg-Injektionslösung in der Fertigspritze oder im Fertig-Pen sowie über ein Pulver zur Herstellung einer Injektionslösung bewerkstelligt werden. In allen 3 Applikationsformen entsprechen 1 ml jeweils 100 mg Mepolizumab.

Studien bestätigen eine gute Wirksamkeit auch unter Real-World-Bedingungen [143–147]. Dabei profitierten nicht nur Patienten mit reiner Typ-2-Inflammation, sondern auch Patienten mit AERD/N-ERD [148]. Das Nebenwirkungsspektrum in der klinischen Routine unterscheidet sich bisher nicht von den Phase-III-Studien. Im Vergleich zu Dupilumab zeigt sich ein anderes Spektrum an unerwünschten Arzneimittelwirkungen [149]. Aufgrund der Zulassung für das Hypereosinophiliesyndrom sowie die EGPA eignet sich Mepolizumab sehr gut auch für CRSwNP-Patienten mit hohen Eosinophilenwerten (> 1500 Zellen/μl) [150]. Zur Indikationsstellung und Verlaufsdokumentation stehen auf Mepolizumab angepasste Dokumentationsbögen zur Verfügung [110, 151, 152].

#### Ausblick auf weitere Biologika

##### Tezepelumab.

Tezepelumab ist ein humaner monoklonaler Antikörper, der TSLP blockiert und somit ein zentrales Alarmin aus dem Epithel hemmt [153]. Die Dosis beträgt 210 mg Tezepelumab alle 4 Wochen s.c. In den USA ist Tezepelumab seit 2021, in der EU seit 2022 für die Behandlung des schweren Asthmas zugelassen [154, 155]. In einer der Zulassungsstudien wurde eine gute Wirksamkeit auch für die komorbide CRSwNP gezeigt [156]. Die Phase-III-Zulassungsstudie WAYPOINT für die CRSwNP konnte signifikante und klinische relevante Ergebnisse für die primären und sekundären Endpunkte gegenüber Placebo zeigen [157]. Von einer Zulassung von Tezepelumab für die CRSwNP noch im Jahr 2025 ist aktuell auszugehen.

##### Depemokimab.

Depemokimab ist ein ultralang wirksamer humaner monoklonaler Antikörper gegen IL‑5, der analog zu Mepolizumab die Bindung von IL‑5 an seinem Rezeptor auf Eosinophilen und anderen Zellen verhindert. Im Unterschied zu den bisher beschriebenen Biologika ist Depemokimab nur alle 6 Monate zu applizieren. Eine Zulassung besteht bisher nicht, doch vielversprechende Studienergebnisse lassen eine Zulassung für das Asthma für 2025 erwarten [158]. Die Phase-III-Zulassungsstudien zur CRSwNP (ANCHOR‑1 und 2) zeigten signifikante positive Ergebnisse für die primären und die meisten der sekundären Endpunkte gegenüber Placebo [159]. Einschränkungen bestehen aus Sicht des Autors des Referates in Bezug auf die klinische Relevanz der statistisch signifikanten Ergebnisse. Eine Zulassung für die CRSwNP im Laufe 2025 bleibt abzuwarten.

##### Benralizumab.

Benralizumab ist ein humanisierter, afucosylierter monoklonaler Antikörper, der gegen die α‑Untereinheit des IL-5-Rezeptors gerichtet ist und durch antikörperabhängige zelluläre Zytotoxizität eine rasche Depletion der Eosinophilen bewirkt. Trotz vielversprechender Ergebnisse in der Phase-III-Zulassungsstudie (OSTRO) für die CRSwNP mit Erreichen der primären Endpunkte erteilte die Food and Drug Administration (FDA) in den USA keine Zulassung, da mehrere sekundäre Endpunkte verfehlt wurden [160]. Auch Beobachtungsstudien zeigten eine Besserung der nasalen Symptome bei Patienten mit eosinophilem Asthma unter Benralizumab-Therapie [161, 162]. Eine weitere Phase-III-Studie (ORCHID) zur CRSwNP verfehlte jedoch die primären Endpunkte, sodass von einer Zulassung von Benralizumab bei CRSwNP nicht auszugehen ist. Abschließende Veröffentlichungen zur Studie liegen bisher nicht vor [163].

Weitere Biologika wie z. B. Lebrikizumab (Anti-IL-13), Itepekimab (Anti-IL-33) und Verekitug (Anti-TSLP-Rezeptor) befinden sich noch in früheren Phasen der klinischen Prüfung, ihre längerfristige Zulassung für die CRSwNP bleibt daher abzuwarten.

## Diskussion

### Standardisierung der Diagnostik

Der enorme Wissenszuwachs zur Pathophysiologie der CRS und die Zulassung der ersten Biologika konnten die Behandlung von Patienten mit schwerer CRSwNP erheblich verbessern. Damit einher ging eine gewisse Standardisierung diagnostischer Werkzeuge zur Indikationsstellung und Verlaufsbeurteilung der Patienten unter Biologikatherapie, aber auch der übrigen CRS-Patienten. Durch den Einsatz einheitlicher diagnostischer Werkzeuge und Scores in vielen der Phase-III-Zulassungsstudien sowie in den internationalen Empfehlungen zur Biologikatherapie haben sich einige Untersuchungen und Scores als eine Art Standard herauskristallisiert, welche den Vergleich von Kohorten sowie eine überregionale Erfassung in Datenbanken und Registern ermöglichen [164, 165]:

- VAS zur Beurteilung der nasalen Symptome sowie des Gesamtbefindens,
- SNOT-22 zur Erhebung der krankheitsspezifischen Lebensqualität,
- Polypen-Score zur Beschreibung des endoskopisch erhobenen nasalen Befundes,
- etablierte Riechtests zur Überprüfung der Riechfunktion,
- Blutuntersuchung (Eosinophile, Gesamt-IgE, ggf. sIgE bei Vorliegen einer möglichen Sensibilisierung oder einer Allergie),
- Lund-Mackay-Score bei Vorliegen einer CT der NNH.

Diese Standardisierung ermöglicht es, trotz aktuell fehlender Head-to-Head-Studien, indirekte Vergleiche der Biologika auf Basis der Zulassungsstudien mit methodischen Einschränkungen anzustellen [166]. Die bisherige Datenlage spricht für eine Überlegenheit von Dupilumab gegenüber Omalizumab, Mepolizumab und Benralizumab, auch wenn letztgenanntes Biologikum die Zulassung für die CRSwNP nicht erhalten wird [167–170]. Alle Biologika zeigen eine gute Wirksamkeit bei geringen Nebenwirkungen [171]. Zudem erlaubt eine einheitliche Diagnostik die Zusammenführung von retrospektiv unter Real-World-Bedingungen erhobenen Daten aus unterschiedlichen Zentren. Beispielhaft seien hier 2 Studien aus Deutschland genannt [119, 122].

Um die Versorgungssituation umfassender zu eruieren, arbeiten die Deutsche Gesellschaft für Hals-Nasen-Ohren-Heilkunde, Kopf- und Hals-Chirurgie und der Ärzteverband Deutscher Allergologen (AeDA) an einem Register für die chronische Rhinosinusitis [172]. Es soll Daten zu Medikation, Komorbiditäten und Krankheitsverläufen erheben, wobei Patienten über eine App eingebunden werden. Langfristig ist eine zentrale Biobank geplant. Eine intelligente IT-Lösung soll zudem die Dokumentation erleichtern und die Anforderungen bei Biologikatherapien absichern. Die Inbetriebnahme ist für Ende 2025 vorgesehen.

### Interdisziplinarität

Patienten mit CRS, insbesondere CRSwNP, leiden häufig an Begleiterkrankungen wie Asthma, Allergien und N-ERD. Aber auch seltenere Erkrankungen wie das Hypereosinophiliesyndrom oder die eosinophile Granulomatose mit Polyangiitis (früher Churg-Strauss-Syndrom) machen eine interdisziplinäre Behandlung notwendig [173]. In einer epidemiologischen britischen Studie zeigte sich eine Prävalenz von Asthma bronchiale bei CRSsNP-Patienten in 21 %, bei CRSwNP-Patienten in 47 % der Fälle im Vergleich zu Kontrollen mit nur 10 % Asthmaprävalenz [174]. Aufgrund dieser Häufigkeit sollte Asthma bei CRS-Patienten standardisiert erfasst und ggf. interdisziplinär behandelt werden. Zur Erfassung der Kontrolle des Asthmas eignet sich z. B. der Asthma Control Test (ACT) sehr gut.

Patienten fordern eine bessere Vernetzung der Fachbereiche, um Komorbiditäten optimal zu behandeln [175]. Dafür sind eine höhere Awareness und Kooperationsbereitschaft zwischen den Disziplinen essenziell [176]. In vielen deutschen Zentren haben sich sog. Typ-2-Inflammationsboards, oft eingebettet in bestehende Boards der an den Standorten vorhanden Allergiezentren, etabliert. Diese ermöglichen eine interdisziplinäre Fallbesprechung und optimierte Therapieentscheidungen. Eine politische Unterstützung solcher Zentren sowie eine Standardisierung und Zertifizierung nach den Kriterien der Deutschen Gesellschaft für Allergologie und klinische Immunologie (DGAKI) wäre daher sinnvoll und wird auch von der Deutschen Gesellschaft für Hals-Nasen-Ohren-Heilkunde befürwortet.

### Individualisierung der Therapie und Biomarker

Während durch die genannten Faktoren eine gewisse Standardisierung im Bereich der Diagnostik erfolgt ist, hat sich therapeutisch durch die Biologika die Möglichkeit einer stärker individualisierten Therapie, basierend auf Endotypen, ergeben [177]. Viele zugrunde liegende Mechanismen sind jedoch noch unzureichend verstanden. Hierzu zählen Umwelteinflüsse wie Viren, Bakterien, Pilze, Allergene, Luftverschmutzung, die Exposition in aktiver oder passiver Form gegenüber Tabakrauch sowie die Exposition gegenüber Noxen am Arbeitsplatz, aber auch klimatische Unterschiede, die von außen auf den Patienten einwirken [177–182]. Auf der anderen Seite stehen das patienteneigene angeborene und erworbene Immunsystem sowie seine individuelle genetischen Prädisposition [4]. Im Bereich der Epithelbarriere treffen diese aufeinander und lösen eine entsprechende Entzündungskaskade aus, wenn die Barriere durchbrochen wird. Bei Gesunden läuft eine spezifische, selbstbegrenzte Immunreaktion ab, die sich gegen Krankheitserreger richtet. Im Gegensatz dazu ist die CRS durch anhaltende, komplexe Immunreaktionen gekennzeichnet, die mit einem „remodelling“ des Gewebes einhergehen können [177]. Obwohl die beteiligten Zellen und Mediatoren der Inflammation vom Typ 1, Typ 2 und Typ 3 weitestgehend bekannt sind, fehlt es an routinetauglichen Biomarkern für eine schnelle Endotypisierung. Potenzielle Biomarker finden sich im Blut (z. B. Eosinophile), Serum (z. B. IgE, eosinophiles kationisches Protein [ECP], „eosinophilic-derived neurotoxin“, Periostin) oder Plasma (z. B. Eotaxin). Im Gewebe können die bereits genannten Biomarker wie Eosinophile, IgE, Periostin und Eotaxin ebenfalls nachgewiesen werden. Auch ist der Gewebenachweis von Interleukinen (z. B. IL‑4, IL‑5, IL-13, IL-33) möglich. Hinzukommen Biomarker wie Interleukine, IgE und Matrixmetalloproteinasen, die lokal im Nasensekret bestimmt werden können, wohingegen ein Bürstenabstrich oder ein „nasal scraping“ darüber hinaus auch zytologische Untersuchungen zulassen [183]. Studien zur mRNA-Expression und Proteinsynthese im Gewebe operierter CRS-Patienten (121 CRSsNP, 134 CRSwNP) zeigten eine Korrelation mit dem klinischen Phänotyp [184, 185]. Zur Endotypisierung wurde IFN‑γ (Typ 1), Charcot-Leyden-Kristall/Galectin-mRNA und ECP (Typ 2) sowie IL-17A (Typ 3) untersucht. Das Vorhandensein (alle Patienten) von Nasenpolypen, komorbidem Asthma und Anosmie war signifikant mit einer Typ-2-Inflammation assoziiert. Die Typ-1-Inflammation kam häufiger bei Frauen vor, und ein purulenter Op.-Situs war mit einer Typ-3-Inflammation korreliert. In der getrennten Analyse (CRSsNP vs. CRSwNP) war die Anosmie in beiden Fällen hinweisend auf eine Typ-2-Inflammation, das Vorliegen von intraoperativem Pus hinweisend auf eine Typ-3-Inflammation [185]. Bachert et al. wiesen bei 84 % von 140 operierten CRSwNP Patienten IL‑5 als Marker für eine Typ-2-Inflammation im Gewebe nach. Bei Vorliegen eines komorbiden Asthmas und > 300 Eosinophilen/μl im Differenzialblutbild war IL‑5 in 98 % der Fälle nachweisbar, wohingegen das Fehlen eines komorbiden Asthmas sowie < 300 Eosinophile/μl nur in 50 % der Fälle mit dem Nachweis von IL‑5 verknüpft war [106]. Trotz fehlender eindeutiger Biomarker machen der Phänotyp der Erkrankung zusammen mit den beschriebenen klinischen Parametern eine grobe Abschätzung des zugrunde liegenden Endotyps schon heute möglich. Dies kann sinnvoll zur Beratung des Patienten in Bezug auf den Verlauf der Erkrankung, das Risiko eines Rezidivs nach Operation, aber auch auf die Erfolgsaussichten einer Biologikatherapie genutzt werden [76, 186]. Da die aktuell zugelassenen Biologika ausschließlich auf die Typ-2-Inflammation abzielen, ist eine grobe Unterscheidung in Typ-2- und Nicht-Typ-2-Inflammation im klinischen Alltag für die Therapieentscheidung ausreichend [3, 4, 13, 107, 187, 188]. Sollten in den nächsten Jahren Therapieansätze auch für andere Entzündungstypen zur Verfügung stehen, muss dies entsprechend berücksichtigt und erweitert werden.

Ist die Indikation zur Biologikatherapie gegeben, stellt sich die Frage nach dem richtigen Biologikum für den individuellen Patienten im Sinne der personalisierten Medizin. Für die überwiegende Anzahl der Patienten wird die Wahl dabei auf Dupilumab fallen. Zum einen, weil die Erfahrung mit Dupilumab aufgrund der Ersteinführung am größten ist, zum anderen, weil es die stärkste Wirkung auf die nasalen Beschwerden zu haben scheint [167–170]. Mechanistisch betrachtet kommt Mepolizumab bei hohen Eosinophilenzahlen, Omalizumab bei zusätzlich vorliegenden IgE-vermittelten Allergien oder beim Vorliegen einer chronischen spontanen Urtikaria in Betracht. Das Vorliegen weiterer Komorbiditäten, die „in-label“ mit einem bestimmten Biologikum behandelt werden können, sollte daher in die Therapieentscheidung mit einfließen (Tab. 2). Ob durch eine solche Auswahl auch eine bessere Wirkung auf die CRSwNP zu erzielen ist, bleibt abzuwarten.

**Tab. 2:** Indikationen und Jahr der Erstzulassung in Deutschland der einzelnen für die chronische Rhinosinusitis mit Nasenpolypen (CRSwNP) zugelassenen Biologika

Indikation	Dupilumab	Omalizumab	Mepolizumab
**Chronische Rhinosinusitis mit Nasenpolypen (CRSwNP)**	*2019*	*2020*	*2021*
**Asthma bronchiale**	*2019*	*2005*	*2015*
**Atopische Dermatitis**	*2017*	*–*	*–*
**Chronisch obstruktive Lungenerkrankung (COPD) mit eosinophilem Phänotyp**	*2024*	*–*	*–*
**Chronische spontane Urtikaria**	*–*	*2014*	*–*
**Eosinophile Granulomatose mit Polyangiitis (EGPA, Churg-Strauss-Syndrom)**	*–*	*–*	*2021*
**Eosinophile Ösophagitis**	*2023*	*–*	*–*
**Hypereosinophiles Syndrom**	*–*	*–*	*2021*
**Prurigo nodularis**	*2022*	*–*	*–*

Vor dem Hintergrund einer Individualisierung der Therapie sowie einer besseren Kosteneffizienz für das Gesundheitswesen wäre die Möglichkeit einer individuellen Anpassung des Therapieintervalls v. a. bei Patienten mit Dupilumab wünschenswert [189]. Aktuell befindet man sich hierbei im Off-Label-Bereich, was entsprechende Konsequenzen für den Verordner mit sich bringen kann und daher nicht zu empfehlen ist. In der einzigen prospektiven Beobachtungsstudie mit 228 Patienten konnte für 3 Viertel der Patienten das Intervall auf mindestens 6–8 Wochen gestreckt werden ohne Einbußen in Bezug auf die Wirksamkeit. Bei 18 % war sogar eine Streckung auf 12 Wochen möglich [126]. Weitere kleinere retrospektive Studien unterstreichen die Machbarkeit einer Verlängerung des Therapieintervalls unter Dupilumab-Therapie [190, 191]. Da Biologika zwischenzeitlich einen relevanten Anteil der Medikamentenkosten im HNO-Bereich ausmachen, wären hierdurch deutliche Einsparungen umsetzbar.

### Neue Therapieziele

Durch die gute Wirksamkeit der Biologika sowie deren möglicherweise krankheitsmodifizierenden Effekt können heute Therapieziele für die CRSwNP formuliert werden, die über eine reine Kontrolle der Symptome hinausgehen und eine Remission bis hin zur Heilung für einige wenige Patienten möglich erscheinen lassen [192–194]. Die Therapieziele und unterschiedlichen Krankheitszustände der CRS wurden hierzu von einer Expertenrunde des European Position Paper on Rhinosinusitis and Nasal Polyps von 2020 (EPOS2020)/des European Forum for Research and Education in Allergy and Airway Diseases (EUFOREA) überarbeitet und für die CRSwNP teilweise neu definiert [108]:

- *Kontrolle:* „Vom Patienten angegebene Kontrolle“. Fehlen klinisch relevanter sinunasaler Symptome einer aktiven Erkrankung (definiert als Schwere der Gesamtsymptome, nasale Obstruktion und Riechvermögen).
- *Remission:* Anhaltende Kontrolle (wie zuvor definiert) für ≥ 12 Monate in Verbindung mit dem Fehlen von Anzeichen einer aktiven Erkrankung, die durch die Nasenendoskopie beurteilt wird. Remission kann mit oder ohne Behandlung erreicht werden (ohne systemische Steroide und/oder Nasennebenhöhlenoperation in den letzten 12 Monaten).
- *Heilung:* Anhaltende Remission ohne Behandlung über mindestens 5 Jahre

Für das Asthma wird ein krankheitsmodifizierender Effekt durch Biologika bereits vermutet [195]. Dies lässt die neu definierten Therapieziele für Typ-2-Erkrankungen wie die CRS in den nächsten Jahren erreichbar erscheinen [196].

Die angesprochenen Themengebiete verdeutlichen, welche großen Fortschritte sich in Teilbereichen der CRS in den letzten Jahren ergeben haben, angefangen von einem tieferen Verständnis der Pathophysiologie und der damit verbundenen Interdisziplinarität über die Standardisierung der diagnostischen Werkzeuge bis hin zur Entwicklung von Therapieverfahren im Sinne der Präzisionsmedizin. Weiterhin bedarf es intensiver Forschungsbemühungen, um die Behandlung von CRS-Patienten mittel- und langfristig zu verbessern. Eine gesellschaftliche Diskussion darüber, wie die begrenzten finanziellen Mittel der Gesundheitssysteme angesichts der enormen Kosten neuer Therapieformen sinnvoll verteilt werden können, ist ebenfalls zwingend notwendig.

## Fazit für die Praxis

- Die chronische Rhinosinusitis (CRS) ist eine heterogene, sozioökonomisch relevante Erkrankung, die mit erheblichen Einschränkungen der Lebensqualität einhergeht.
- Trotz immenser Fortschritte in Bezug auf die Pathophysiologie, einer Vereinheitlichung der Diagnostik sowie der Einführung von Biologika bei schwerer chronischer Rhinosinusitis mit Nasenpolypen (CRSwNP) hat sich an der leitliniengerechten Behandlung der Mehrzahl der Patienten nichts geändert.
- Nasenspülungen mit isotoner Kochsalzlösung und intranasale Glukokortikosteroide (nGKS) stellen die medikamentöse Standardtherapie dar.
- Bei fehlendem Ansprechen schließt sich eine endoskopische Nasennebenhöhlenoperation (ESS) an die sich am Ausmaß der Erkrankung orientiert.
- Alternativ kann bei chronischer Rhinosinusitis ohne Nasenpolypen (CRSsNP) eine antibiotische Langzeittherapie, bei CRSwNP der Einsatz von systemischen Glukokortikosteroiden (sGKS) erwogen werden.
- Kommt es durch die ESS zu keiner ausreichenden Symptomkontrolle, besteht die Möglichkeit einer Revisions-Op. oder des Einsatzes von Biologika.
- Bei durch nichtsteroidale Antirheumatika (NSAID) exazerbierter Atemwegserkrankung (N-ERD) ergibt sich ergänzend die Möglichkeit einer Aspirin-Dauertherapie nach adaptiver Desaktivierung („aspirin-therapy after desensitization“, ATAD).
- Die Biologikatherapie sollte indikationsgerecht verordnet und gut dokumentiert werden.
